# Testing the passive sampling hypothesis: The role of dispersal in shaping microbial species-area relationship

**DOI:** 10.3389/fmicb.2023.1093695

**Published:** 2023-01-26

**Authors:** Wei Deng, Guo-Bin Yu, Xiao-Yan Yang, Wen Xiao

**Affiliations:** ^1^Institute of Eastern-Himalaya Biodiversity Research, Dali University, Dali, Yunnan, China; ^2^Collaborative Innovation Center for Biodiversity and Conservation in the Three Parallel Rivers Region of China, Dali, Yunnan, China; ^3^The Provincial Innovation Team of Biodiversity Conservation and Utility of the Three Parallel Rivers Region, Dali University, Dali, Yunnan, China; ^4^International Centre of Biodiversity and Primates Conservation, Dali, Yunnan, China; ^5^Yunling Black and White Snub-Nosed Monkey Observation and Research Station of Yunnan Province, Dali, Yunnan, China

**Keywords:** biodiversity patterns, rare species, species replacement, microbial dispersal, passive sampling hypothesis

## Abstract

Dispersal is one of the key processes determining biodiversity. The passive sampling hypothesis, which emphasizes dispersal processes, suggests that larger habitats receive more species from the species pool as the main mechanism leading to more species in larger habitats than in smaller habitats (i.e., species-area relationships). However, the specific mechanisms by which dispersion shapes biodiversity still need to be discovered due to the difficulties of quantifying dispersal and the influence of multiple factors. Solving the above problem with a designed experiment is necessary to test the passive sampling hypothesis. This study designed a passive sampling experiment using sterile filter paper to quantify the microbial diffusion process, excluding the effects of pure sampling effects, habitat heterogeneity, and extinction processes. The results of high-throughput sequencing showed that a larger filter paper could receive more colonists, and the passive sampling hypothesis of SAR was confirmed. Dispersal shaped SAR by increasing species richness, especially rare species, and increasing the species replacement rate between habitats. These two processes are the mechanisms by which dispersal shapes biodiversity patterns. Compared with the results of this study, the commonly used mathematical model of passive sampling was able to predict the richness of non-rare species accurately but underestimated the richness of rare species. Underestimating rare species by mathematical models of passive sampling is more severe in small habitats. These findings provide new insights into the study of dispersal processes and the mechanism of species-area relationships.

## 1. Introduction

Biodiversity patterns are at the heart of ecological research. Ecologists have developed dozens of ecological hypotheses to answer the critical question of which processes lead to spatial and temporal changes in biodiversity patterns (Holyoak et al., 2005; Verhoef and Morin, 2010; Morin, 2011; Scheiner and Willig, 2011). Many hypotheses emphasize the importance of one or more abiotic factors (e.g., climate, disturbance) and biological factors (e.g., competition, predation; Vellend, 2016). Despite being complicated, these elements are controlled by high-level mechanisms determining biodiversity. Ecologists have been working to find a deep unifying theory (McGill, 2010; Vellend, 2016). MacArthur and Wilson (1967) theorized island biogeography and argued that the equilibrium between dispersal and extinction determines regional biodiversity. MacArthur (1969) further emphasized the role of speciation soon after. On this basis, Vellend (2010) proposed the theory of ecological communities, arguing that speciation, dispersal, ecological drift, and selection are vital processes that determine biodiversity (ecological drift and selection ultimately play a role through extinction). In summary, the fundamental determinants of biodiversity patterns are the three critical processes of speciation, dispersal, and extinction. Current research does not understand enough about those three process, resulting in the pattern of biodiversity and its formation mechanism is still unclear.

Of these three fundamental processes, speciation and dispersal are how communities acquire species, while species extinction is how communities lose species. Thus these processes can be summarized as biodiversity = speciation + dispersal–extinction (Ricklefs and Schluter, 1993). Dispersal is the only factor leading to an increase in regional biodiversity other than speciation (Vellend, 2016). The speciation process is prolonged, and its impact on diversity can be negligible in a short time. Although ecologists have long recognized the role of dispersion in the community assembly process, it was not until the development of the meta-community theory that the process of dispersion was recognized as one of the determinants of biodiversity (Leibold et al., 2004). Through dispersion, local-scale biodiversity is connected to regional biodiversity, and regional biodiversity is connected to global biodiversity. Thus diffusion is a bridge to unify biodiversity studies from the local to the global scale (Leibold et al., 2004; Vellend, 2016). For this reason, deciphering the critical process of dispersal is critical for understanding biodiversity patterns.

The species-area relationship (SAR), which describes the positive correlation between species richness and habitat area, is considered the first biodiversity pattern and a vital tool for biodiversity assessment and conservation strategies (Arrhenius, 1921; MacArthur and Wilson, 1963, 1967; Lomolino, 2000; Matias et al., 2014). There are many hypotheses for SAR formation mechanisms. The island biogeographic equilibrium hypothesis (also known as the area *per se*) suggests that large islands have low random extinction rates; the habitat heterogeneity hypothesis suggests that large islands have more habitat types; moreover, the passive sampling hypothesis suggests that large islands randomly collect more individuals (Connor and McCoy, 1979; Rosenzweig, 1995; With et al., 1996). These three hypotheses are the three most widely accepted mechanisms. In addition, disproportionate effects (including the disturbance and environmental filtering hypotheses), which consider disturbance and environmental filtering more potent on small islands, are also gaining attention (Chase et al., 2019; Gooriah et al., 2020; Liu et al., 2020). However, these hypotheses are not mutually exclusive. In ecosystems where they often act together, it is challenging to distinguish their relative contributions and independently test each hypothesis’s independent role (Rosenzweig, 1995). Connor and McCoy (1979) proposed the passive sampling hypothesis based on oceanic islands, arguing that larger islands can effectively receive more colonists than smaller one. This dispersion process is key to determining SAR. Coleman (1981) and Coleman et al. (1982) developed this theory from a mathematical perspective and proposed a mathematical model for predicting island species richness based on the passive sampling hypothesis. If the passive sampling results predicted by Coleman’s model are considered as the null hypothesis and the remaining hypotheses as alternative hypotheses, the relative roles between the SAR formation mechanisms can be relatively distinguished when comparing the observed data with the predicted data (With et al., 1996). Therefore, the passive sampling hypothesis emphasizing diffusion becomes one of the essential formation hypotheses of SAR.

However, the extent to which this hypothesis explains biodiversity has been controversial, with some studies providing evidence to support it (Hill et al., 1995; Ouin et al., 2006; Bidwell et al., 2014; Gooriah and Chase, 2020) and many arguing that it is invalid (Bolger et al., 1991; Wang et al., 2010; Xu et al., 2017). Due to the following difficulties, experiments have yet to directly confirm the shaping of SAR by this unique dispersal process. (1) Dispersal is often challenging to quantify. Current studies can only conduct qualitative studies representing dispersal using dispersal-related properties, such as geographic distance and connectivity (Nathan, 2001). (2) Pure sampling effects often mask dispersal. When conducted based on successively nested regions, the increase in species richness over the area due to incomplete sampling (i.e., pure sampling effect) is confused with dispersal, making the passive sampling hypothesis appear invalid (Hill et al., 1995; Deng et al., 2022a). (3) The role of dispersal is difficult to disentangle from the not mutually exclusive mechanisms that shape SAR. The dispersal process appears to involve the movement of organisms in only two locations, but the consequences of this movement are very complex (Leibold et al., 2004; Holyoak et al., 2005; Haegeman and Loreau, 2014). After dispersion, the community may be adjusted by the extinction process, which makes it difficult to distinguish the effects of dispersion and extinction in natural communities (Deng et al., 2022b).

The passive sampling hypothesis is based on two assumptions: 1. Islands sample individuals randomly and independently. In other words, interspecific or intraspecific forces do not modify the probability of individual occurrence; 2. Larger habitat areas have higher migration rates and, thus, more species (area effect of dispersal; Connor and McCoy, 1979; With et al., 1996). It is necessary to verify the passive sampling hypothesis by avoiding the influence of the extinction process through controlled experiments, completely excluding the sampling effect, distance attenuation relationship, and spatial heterogeneity, and quantitatively verifying the area effect of dispersal.

This study established a passive sampling experiment using sterile filter paper. A fixed area sterile qualitative filter paper is exposed to a regional microbial species bank for 12 h, followed by 16S and ITS amplicon sequencing of the entire filter paper. The SAR curves were plotted by combining the filter papers into a model with seven groups of islands of the gradually increasing area and compared with the random sampling model proposed by Coleman (1981). In the experiment, we sequenced the whole filter paper to obliterate the effect of the sampling effect; the homogeneous filter paper avoided the effect of habitat heterogeneity. The effects of the extinction process should have been minimized or negligible in this model. Because of the short period of the experiment, the dispersion process should be the determinant of biodiversity in the filter paper island model. We look forward to discovering larger filter islands that will receive more colonists from the regional seed bank, resulting in significant SARs.

## 2. Materials and methods

### 2.1. Establishment of the experimental system

This study used sterile filter paper to establish the microcosm system. The filter paper should be a manageable size to be sequenced and not too large to sequence the whole filter paper and cause incomplete biodiversity monitoring. After pre-experimental exploration, 60 × 60 filter paper can meet these requirements while ensuring aseptic operation. In order to form an area gradient, filter papers of the same area were combined in this study to obtain an island model.

Take eight pieces of 60 cm × 60 cm qualitative filter paper for autoclaving at 121.3°C for 30 min. Soak sterile filter paper in 75% medical alcohol for later use. Take 3.4 m × 3.4 m wooden planks and disinfect them with 75% medical alcohol. Use sterile kraft paper to cover the surface of the board. Eight pieces of filter paper were taken out of the alcohol, flattened, fixed on the wooden board with sterile thumbtacks, and exposed to the open and unsheltered test land (100.1738E, 25.6844 N) in the Cangshan Erhai National Nature Reserve in Yunnan, China for 12 h (Placement of 3 rows and 3 columns, 50 cm interval between filter papers).

### 2.2. Sample collection

Place the filter paper in a sterile sealed bag and send it immediately to the laboratory for processing. Add 25 ml of PBS buffer (pH = 8) to the sample bag, then sonicate for 1 min, vortex for 10 s, repeat the ultrasonic wash, and vortex once. Collect the wash solution in a 50 ml sterile centrifuge tube, re-add 25 ml of PBS buffer to the sample, and repeat the above steps. Combine the two washes in a 50 ml sterile centrifuge tube, centrifuge 13,000 g for 10 min, and collect centrifugal precipitation. The resulting centrifugal precipitation was quick-frozen on dry ice and placed in an ultra-low temperature freezer at −80°C for backup.

### 2.3. Microbial analyses

Microbial DNA was extracted from filter paper samples using the EZNA® Soil DNA Kit (Omega Biotek, Norcross, GA, United States) according to the manufacturer’s protocols. For bacteria, we targeted the V3-V4 region of the 16S ribosomal RNA (rRNA) gene, using the 338F (5′-ACTCCTACGGGAGGCAGCAG-3′) and 806R (5′-GGACTACHVGGGTWTCTAAT-3′) primer pairs (Wang et al., 2019). For fungi, we targeted the ITS1-1F region of the nuclear ribosomal internal transcribed spacer region (ITS) gene, using ITS1-1F-F (5′-CTTGGTCATTTAGAGGAAGTAA-3′) and ITS-1F-R (5′-GCTGCGTTCTTCATCGATGC-3′; Manter and Vivanco, 2007). PCRs were performed in triplicate in a 20 μl mixture containing 4 μL of 5× FastPfu Buffer, 2 μL of 2.5 mM dNTPs, 0.8 μL of each primer (5 μM), 0.4 μL of FastPfu Polymerase and 10 ng of template DNA. The PCR program for the 16S rRNA gene was as follows: 3 min of denaturation at 95°C; 27 cycles of 30 s at 95°C, 30 s of annealing at 55°C, and 45 s of elongation at 72°C; and a final extension at 72°C for 10 min. For the ITS1-1F region, the PCR program was as follows: samples were initially denatured at 98°C for 1 min, followed by 30 cycles of denaturation at 98°C for 10 s, primer annealing at 50°C for 30 s, and extension at 72°C for 30 s. A final extension step of 5 min at 72°C was added to ensure complete amplification of the target region. The resulting PCR products were extracted from a 2% agarose gel, further purified using the AxyPrep DNA Gel Extraction Kit (Axygen Biosciences, Union City, CA, United States), and quantified using QuantiFluor™-ST (Promega, United States) according to the manufacturer’s protocol.

Purified amplicons were pooled in equimolar amounts and paired-end sequenced (2 × 300) on an Illumina NovaSeq platform (Illumina, San Diego, United States) according to standard protocols. Sequencing depth is higher than 50,000 sequences. The analysis was conducted following the “Atacama soil microbiome tutorial” of QIIME2 docs and customized program scripts^1^ (Bokulich et al., 2018). Briefly, raw data FASTQ files were imported in an appropriate format for the QIIME2 system using the QIIME tools import program. Demultiplexed sequences from each sample were quality filtered, trimmed, denoised, and merged. Then, the chimeric sequences were identified and removed using the QIIME2 DADA2 algorithm plugin to obtain the feature table of amplicon sequence variants (ASVs; Callahan et al., 2016). The QIIME2 feature-classifier plugin was then used to align ASV sequences to the pretrained GREENGENES 13_8 99% database (trimmed to the V3-V4 region bound by the 338F/806R primer pair for bacteria) and UNITE database (for fungi) to generate the taxonomy table (Bokulich et al., 2018). Any contaminating mitochondrial and chloroplast sequences were filtered using the QIIME2 feature-table plugin.

### 2.4. Data analysis

Plot rarefaction curves using the QIME2 core-diversity plugin (Bokulich et al., 2018). Rank Abundance data should be calculated using the Biodiversity R package in R 4.2.1 following the ASV table (Kindt and Coe, 2023), and the Rank Abundance curve should be shown using ggplot2 (Wickham, 2017).

After Z-score standardization of the relative abundance of ASV tabular data annotated to the genus level, the grouping cluster heat map was drawn using the pheatmap package in R 4.2.1 to show the 20 genera with the highest relative abundance (Kolde, 2019).

**Figure 1:**
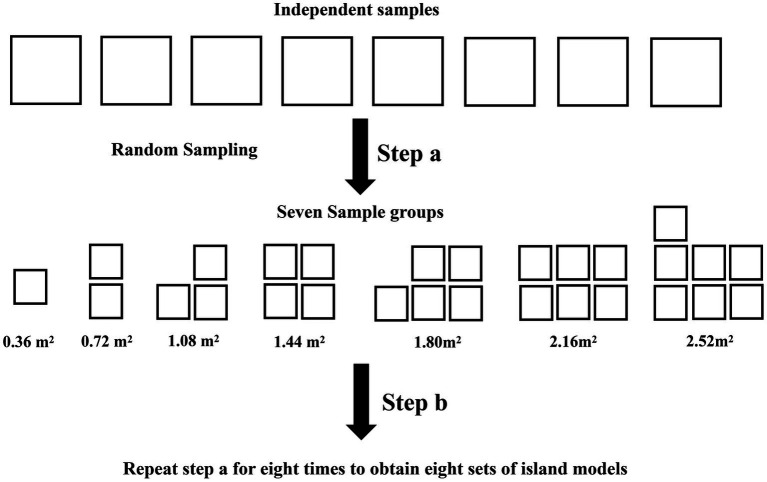
Schematic graph of sampling. Each box in the graph represents one filter paper. The area of each independent sample is 0.36 m^2^. Perform Step a to obtain seven sample group. Perform step b eight times to obtain eight island models, each containing seven sample groups.

The microbial species richness of each sample was calculated using the vegan package in R 4.2.1 (Dixon, 2003). To execute random sampling from eight filter paper samples, use the sample function in R 4.2.1. Allow replicates to randomly sample 1 to 7 samples and superimpose the filter paper areas to obtain 7 sample groups with areas of 0.36, 0.72, 1.08, 1.44, 1.80, 2.16, and 2.52 m^2^ (Figure 1). Follow the above method eight times to obtain eight sets of island models. After logarithmic transformation of the species richness and area, SAR is constructed using a linear model in log–log space with:


$$\log\left(S\right)=C+z\times \log\left(A\right)$$

S is species richness, A is the area, C and z are fitting parameters, where z is slope (Arrhenius, 1921). log(A) represents the area, which we expect to be positive. Therefore we use square centimeters as the unit of area for our calculations. Plot and perform curve fitting using R 4.21, statistically fitting parameters. A total of 8 SAR curves were obtained.

Amplicon sequence variants with total relative abundance greater than 0.1% of the eight samples were defined as abundant species, less than 0.01% rare species, and those between 0.1 and 0.01% moderate species. The passive sampling hypothesis imagines individuals as darts and islands as targets, with different colored darts representing different species. When darts are thrown randomly at the target, larger islands accumulate more darts and thus more species (Connor and McCoy, 1979; Coleman, 1981; Coleman et al., 1982; With et al., 1996). Coleman (1981) described this process as the following equation:


$$E\left(Sj\right)=\overset{S}{\underset{i=1}{\sum }}1-{\left(1-\frac{aj}{AT}\right)}^{ni}$$

E(Sj) is the expectation of the number of species under the area of island j. aj is the area of the jth island, AT is the sum of the areas of all islands under study, and ni is the sum of the richness of species occurring on island j over all islands. The inner term of the summation symbol is the probability of species i was occurring on the island. Given ni, darts are thrown at the target, and the expected number of species on the island is obtained when all species are added up. Passive sampling effects occur when actual monitoring results are consistent with this model. The numbers of abundant, rare, and moderately abundant species were predicted separately for different filter paper areas concerning Coleman (1981) random sampling model. The model predictions were subjected to a paired-sample t-test with actual observations, and the variance test was performed at the 95% confidence level.

Calculate the sample unweighted UniFrac and weighted UniFrac indices using the phyloseq packet in R 4.2.1 for assessing microbial community differences (McMurdie and Holmes, 2013). The weighted Unifrac distance mainly represents the difference between groups of highly enriched species, while the unweighted Unifrac distance better represents the difference between groups of rare species (Lozupone and Knight, 2005). Use corrplot to create a heat map showing the unweighted UniFrac and weighted UniFrac indices between samples (Wei and Simko, 2021).

Beta diversity partition of eight samples using ade4, adegraphics, and adespatial packages in R 4.2.1 and presentation of the results in Triangular plots (Dray and Dufour, 2007; Carvalho et al., 2013; Legendre, 2014; Bougeard and Dray, 2018; Thioulouse et al., 2018; Guénard and Legendre, 2022).

## 3. Results

### 3.1. Microbial diversity

The extracted DNA template content is insufficient to fulfill the PCR criteria since there is not enough fungal biomass on the filter paper, and the ITS sequence sequencing is unsuccessful. The 16S rDNA PCR product was tested, and the sequencing quality was qualified, so this study only discussed bacteria.

The eight filter paper samples obtained 492,880 16 s rDNA sequences, of which 234,546 high-quality sequences were obtained after quality filtering and denoising. Eight filter paper samples showed a rapid smoothing of the rarefaction curve, indicating that our sampling and sequencing were reasonable (Supplementary Figure S1). A total of 7,895 ASVs were assigned to 2 kingdoms, 43 phyla, 121 classes, 201 orders, 276 families, 500 genera, and 295 species. Detailed classification list see Supplementary Table S1.

**Figure 2:**
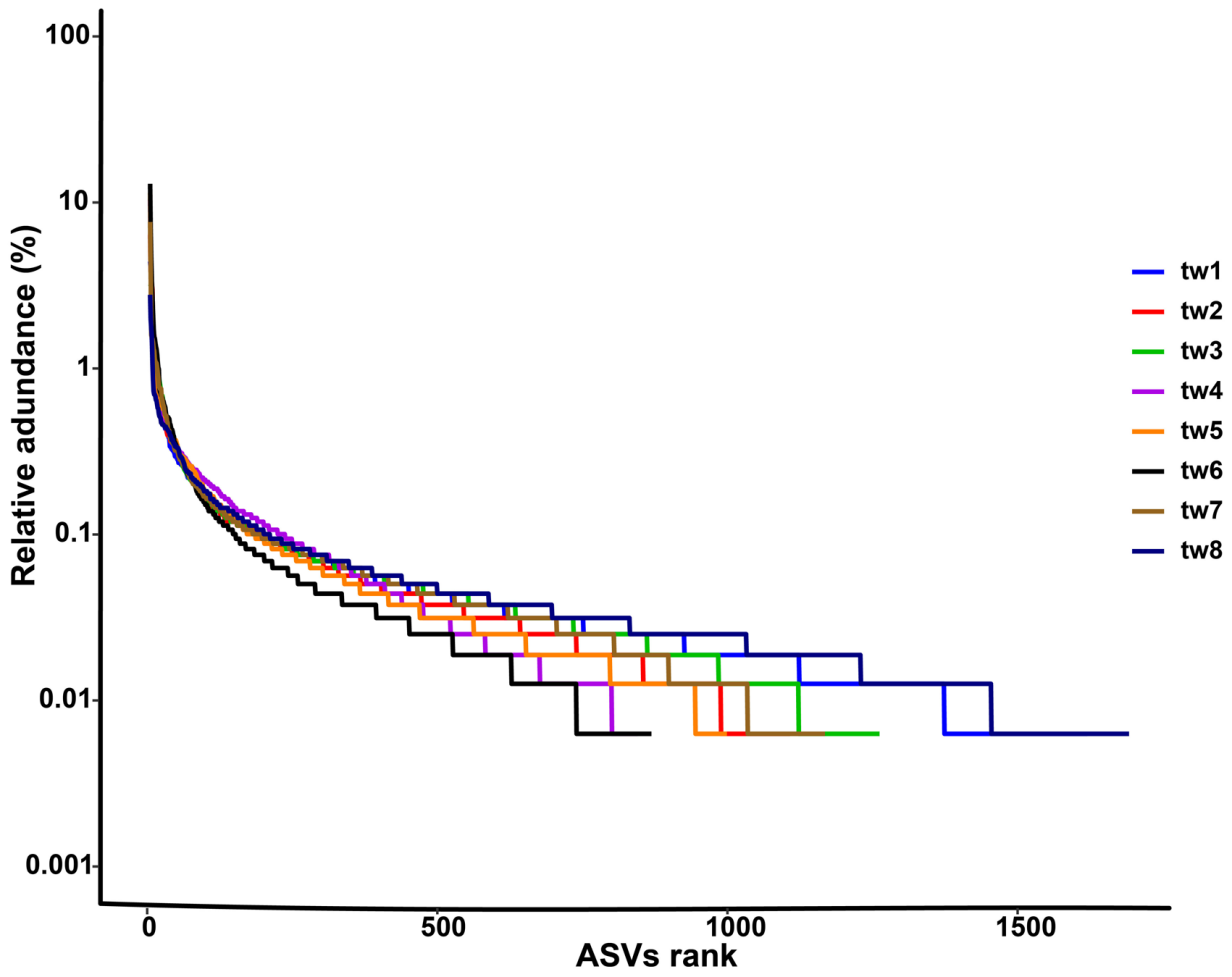
Rank abundance curve of each samples. The sample ids for the filter paper are tw1 through tw8.

The richness rank curves showed that there were 145 abundant species with a richness greater than 0.1% in the bacterial communities of the eight samples, and each sample contained a total of 5,853 rare species with a richness of less than 0.01% (Figure 2). Since all species in this study are from dispersal, the rarity of the species represents the strength of the dispersal ability.

The ASV numbers (i.e., Richness) of the eight samples ranged from 766 to 1,546, and the alpha diversity indices of each sample are shown in Table 1.

**Table 1:** The alpha diversity index of each sample.

Sample ID	Shannon	Simpson	Richness	ACE
tw1	6.36	0.99	1461.00	17.08
tw2	5.71	0.99	999.00	14.84
tw3	6.07	0.99	1144.00	15.67
tw4	5.49	0.98	782.00	13.93
tw5	5.49	0.98	924.00	14.16
tw6	4.98	0.97	766.00	13.04
tw7	5.89	0.99	1066.00	15.25
tw8	6.60	1.00	1546.00	17.39

**Figure 3:**
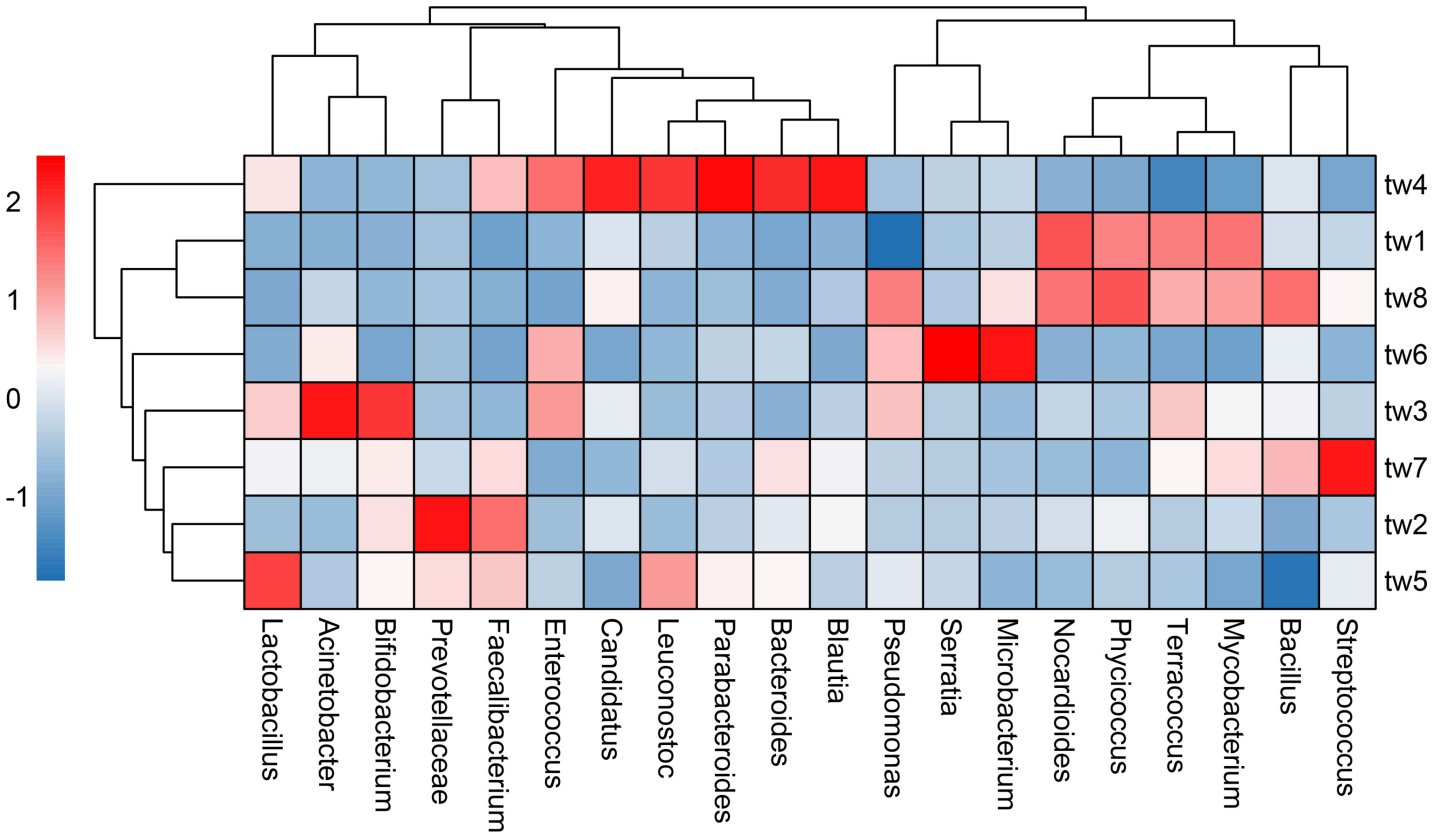
Heat map of relative abundance clustering of dominant genera. The tree diagram is obtained based on similarity clustering. The sample ids for the filter paper are tw1 through tw8.

Proteobacteria, Actinobacteria, and Firmicutes were the dominant phylum common to all samples. Streptococcus, Bacillus, Mycobacterium, Terracoccus, Phycicoccus, Nocardioides Microbacterium, Serratia, Pseudoalteromonas, Blautia, Bacteroides, Parabacteroides, Leuconostoc, Candidatus Solibacter, Enterococcus Faecalibacterium, Prevotellaceae, Bifidobacterium, Lactobacillus, and Acinetobacter were the dominant genera in the top 20 relative abundance of each sample. The relative abundance of each genus varied widely among samples (Figure 3).

### 3.2. Dispersal shaped significant species-area relationship

**Figure 4:**
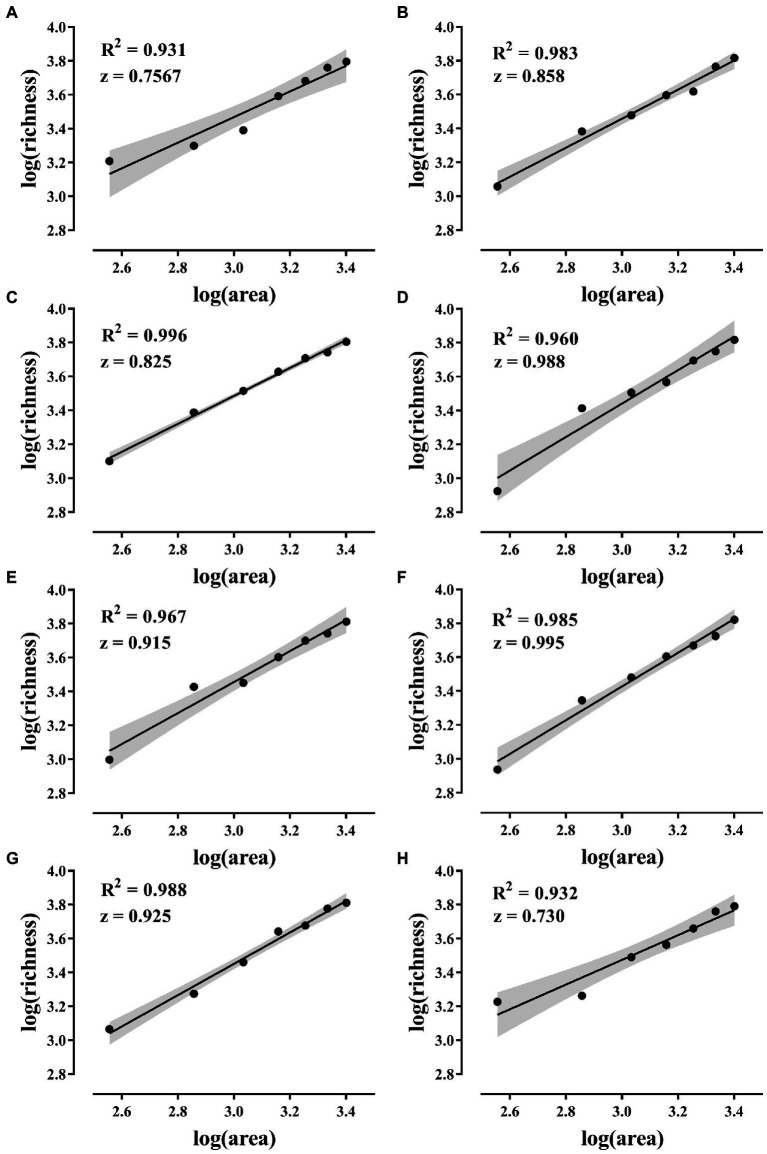
SAR curves. **(A–H)** The SAR curves of the eight island models in Figure 1. The solid line in the figure shows the fitted curve. SAR was constructed using a linear model in log–log space with log(S) = C + z * log(A) (S is species richness, A is area, C and z are fitting parameters, where z is the slope). The gray areas are 95% confidence intervals and the *p*-values of the fitted curves are all less than 0.001.

All eight combinations had significant SAR curves, and the average slope of each SAR curve was 0.874 (Figure 4).

### 3.3. High rates of species replacement due to dispersal processes are a major component of beta diversity

**Figure 5:**
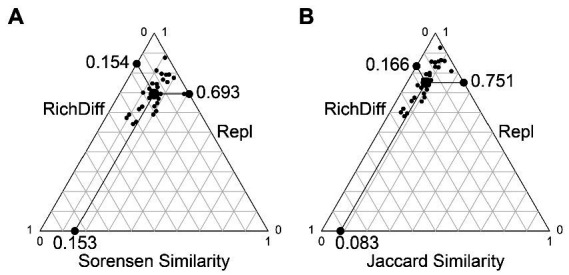
Beta diversity partition results. **(A)** The beta diversity partition based on the Sorensen index. **(B)** The beta diversity partition based on the Jaccard index. Each black dot represents a pair of samples. Their positions were determined by a triplet of values from the species composition similarity (Similarity), species replacement (Repl), species richness difference component (RichDiff); each triplet sums to 1. The large circular dot in each graph is the centroid of the points; the larger black dots represent the mean values of the Similarity, Repl, and RichDiff components. The line between the three edges in the figure is the scale of the coordinate axis, each scale is 0.1.

The results of the beta diversity partition show that beta diversity is mainly contributed by replacement. Based on the Sorensen and Jaccard indices, substitution contributed 81.8 and 91.9% of beta diversity, respectively; Richness differences (loss and increase of species) contributed only 18.2 and 8.1% of beta diversity, respectively (Figure 5).

### 3.4. Rare species are major contributors to biodiversity

**Figure 6:**
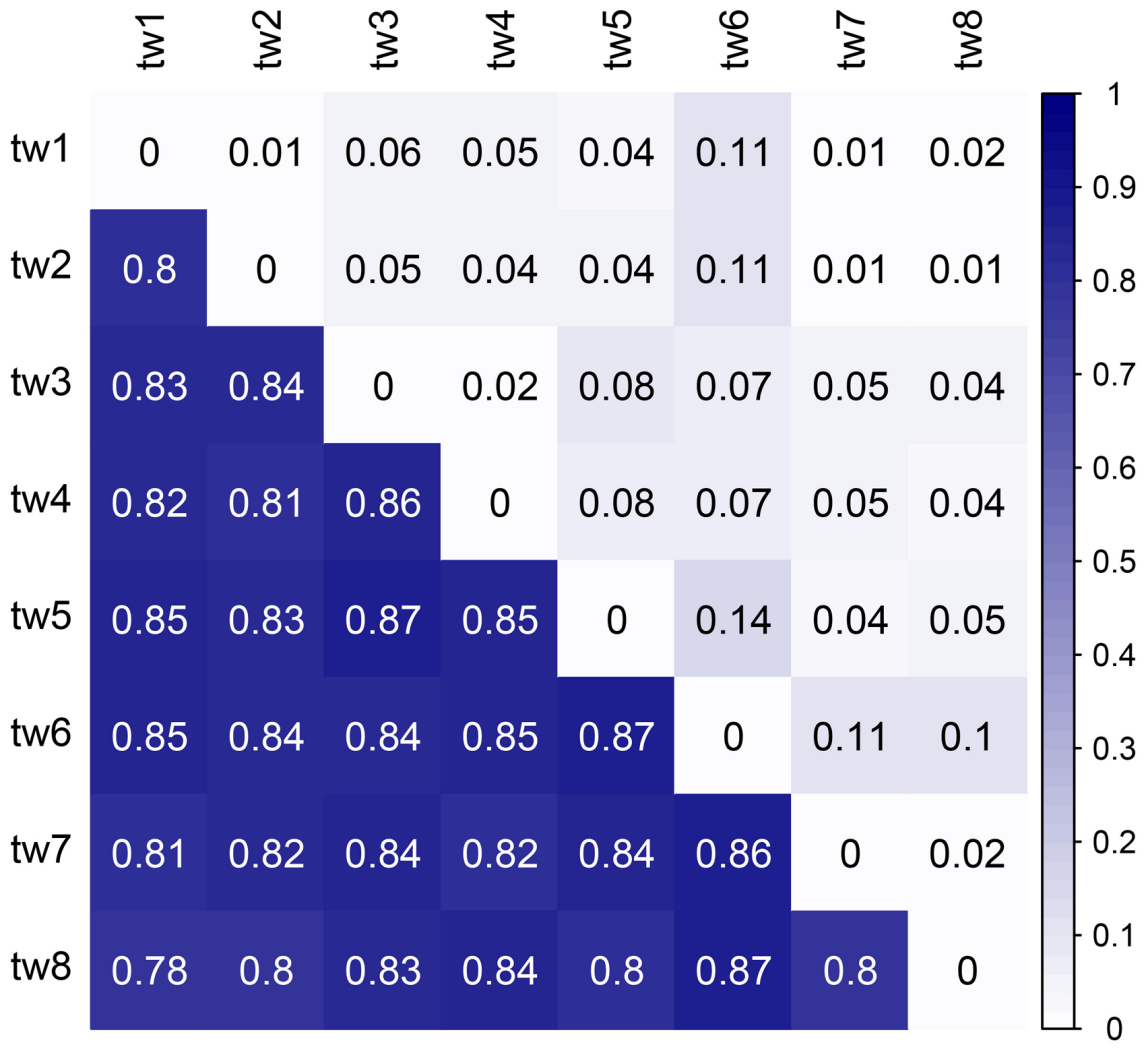
Heat map of Unifrac distances between samples. The upper triangle is the weighted Unifrac distance, and the lower triangle is the unweighted Unifrac distance. Unifrac distance combines community composition and phylogenetic distance. The weighted Unifrac distance also considers species richness, while the unweighted Unifrac distance only considers the presence or absence of species. Therefore, the unweighted Unifrac distance emphasizes rare species, and the weighted Unifrac distance has a low contribution of rare species.

The beta diversity was high among the samples, with unweighted Unifrac distances above 0.78, showing a considerable variation in microbial communities. However, the weighted Unifrac distances were low; none exceeded 0.15 (Figure 6).

### 3.5. Passive sampling mathematical models underestimate the richness of rare species

Species richness was predicted using Coleman (1981) formula and compared with the observed data obtained in this study. The results showed that observations of abundant and moderate species did not differ significantly from the random sampling model (Table 2). In contrast, observations of rare species differed significantly from the random sampling model. The smaller the habitat area, the greater the difference between the species richness observed for rare species and the random sampling model. The random sampling model underestimated the richness of rare species in this study.

**Table 2:** Differences between species richness predicted by Coleman’s random sampling model and actual observed richness.

Area	Abundant species richness	Abundant species expectation	Moderate species richness	Moderate species expectation	Rare species richness	Rare species expectation
0.36	117	116.99	428	408.81	1,071	344.64
0.72	126	126	550	547.52	1,317	845.93
1.08	132	132	589	588.82	1736	1318.16
1.44	142	142	838	837.99	2,924	2419.99
1.80	144	144	920	919.99	3,752	3375.47
2.16	144	144	1,075	1074.99	4,532	4253.83
2.52	145	145	1,181	1180.99	4,924	4808.07
Mean of differences	−0.00143		−3.127		−412.8	
SD of differences	0.00378		7.141		190.5	
*t*	1		1.159		5.735	
*p*	0.3553		0.2907		0.0012**	

** Indicates that *p* value is less than 0.01.

## 4. Discussion

In this study, 60 × 60 cm filter paper was used to comprehensively monitor the bacterial diversity of the entire filter paper by high-throughput sequencing method. The results show that the sequencing of this study is reasonable (Supplementary Figure S1). In addition, the filter paper had high bacterial alpha diversity with dominant phyla consistent with the dominant taxa in natural habitats, demonstrating that the bacteria on the filter paper were randomly collected from the regional species pool (Table 1; Figures 2, 3). Therefore, this study successfully constructed an island model of passive sampling from the regional species pool, which fully met the conditions for validating the passive sampling hypothesis.

The results showed that all eight island model combinations had significant SAR. That is, larger habitats can receive more species from regional species pool. The area effect of dispersal and the passive sampling hypothesis of SAR was confirmed (Figure 4). The slope of the SAR curve in this study far exceeds that of previous studies and exceeds the practical value of 0.25 slope for plants and animals (Horner-Devine et al., 2004; Deng et al., 2021). Previously, we conducted experiments in the open microcosm system to test the passive sampling hypothesis. However, the 15-day experimental period allowed the change of the experimental system to undergo an extinction process after the area effect of dispersion occurred (Deng et al., 2022a). Dispersal and extinction play opposite roles in determining the biodiversity of an area. The combined effect of the two can obscure SAR. The micro-island system in this study was exposed to the species bank for only 12 h, minimizing the impact of extinction. Therefore, focused dispersal became the key to confirming this study’s SAR passive sampling hypothesis. Many previous studies have found that the slope of microbial SAR is generally low (Horner-Devine et al., 2004). Researchers believe that microorganisms have individual smallness, short generation, wide distribution, dormancy, and tolerance to extreme environments, and the biodiversity pattern is usually weak (Prosser et al., 2007; Meyer et al., 2018). This study found that strong patterns of microbial diversity are related to the following factors.

First, dispersal brings different species compositions to the same habitat area, and the resulting higher replacement rate is the cause of high slope SAR (Cadotte, 2006). The results of the beta diversity partition showed that replacement was the main component of beta diversity, and the contribution degree of species loss and increase was less than 20% (Figure 5). The higher contribution of replacement rates means that although there is little difference in the number of species captured in the same habitat area, the species composition between samples is very different. When these habitat areas are superimposed, total species richness increases dramatically. Therefore, the initial process of dispersion, the movement of species in space, will bring significant structural differences to communities. This process is also essential for dispersion to shape biodiversity, especially beta diversity (Howeth and Leibold, 2010).

The primary mechanism responsible for the high species replacement rate is rare species brought about by dispersal. The Rank abundance curve shows that the rare species in each sample in this study account for a large proportion and are the main component of the total species richness (Figure 2). At the same time, the unweighted Unifrac distance of the 8 samples was higher, while the weighted Unifrac distance was lower (Figure 6). The weighted Unifrac distance mainly represents the difference between groups of highly enriched species, while the unweighted Unifrac distance better represents the difference between groups of rare species (Lozupone and Knight, 2005). These findings suggest that the species composition of the eight samples is highly different and that rare species are the main taxa responsible for this difference. According to this, it can be considered that rare microbial species contribute greatly to the shaping of diversity patterns (Nolte et al., 2010; Lynch and Neufeld, 2015; Bay et al., 2020). Species richness was predicted using the random sampling model of Coleman (1981) and compared with the observed data obtained in this study. The results showed that the filter paper model for abundant and moderate species was entirely consistent with the random sampling model (Table 2). However, this model underestimated the number of rare species. The Coleman model had even lower predictive power for rare species in small habitats (Table 2). The Coleman model presupposes that each species has the same probability of being sampled by an island per unit area. However, 6,178 rare ASVs that appeared on only 1 filter paper were present in this study. These ASVs occurred with a lower probability than the abundant species, which may be why rare species do not fit the Coleman model. However, these results suggest that the passive sampling process can shape significant SARs even if species have different probabilities of occurrence, which further extends the application of the passive sampling hypothesis. It also further reminds us that using the Coleman model to predict passive sampling results in actual survey data is reasonable. However, it requires special attention to rare species in small habitats.

Unlike previous studies, this study used the DADA2 algorithm to process sequencing results into higher-resolution ASV instead of OTU to assess microbial diversity. ASV can recognize differences in single nucleotides and is more sensitive to identifying rare species (Callahan et al., 2016). This should be one of the reasons why this study was able to find high-slope SAR. There are many main processing algorithms for high-throughput sequencing data of microorganisms. These algorithms have different sensitivity to rare species identification, and this difference may lead to differences in the number of rare species monitored (Nearing et al., 2018; Jia et al., 2022), resulting in changes in diversity patterns such as SAR. Future research on microbial diversity patterns should pay special attention to the importance of rare species, and the impact of different sequence processing algorithms on microbial diversity patterns should also be studied.

The results of this study also show that a return to the three critical processes of speciation, dispersal, and extinction through design experiments can verify the formation mechanism of SAR one by one, thereby helping to elucidate the critical question of biodiversity patterns (Deng et al., 2022b). Although SAR is known as the first pattern of biodiversity, its formation mechanism is not clear. There are many hypotheses used to explain SAR, but the independent role of these hypotheses has not been proven individually (Connor and Simberloff, 1979; Connor and McCoy, 2013). The unique properties of microorganisms facilitate the experimental study of three fundamental processes. The microbial generation is short, the community dynamics change rapidly, and the community starting point can be controlled, which is especially suitable for quantitatively carrying out extinction and dispersal dynamic experiments on the temporal and spatial scale (Deng et al., 2022b). Microbial genomes are simpler and more variable than plants and animals and are expected to explore speciation’s role on biodiversity patterns in shorter time scales (Chase et al., 2021). Future biodiversity pattern research can be based on microbial microcosmos experiments, which are expected to indeed clarify the spatial and temporal changes of biodiversity patterns from the perspective of three fundamental processes speciation, dispersal, and extinction.

## 5. Conclusion

Larger habitats can receive more species from regional species pool, and the passive sampling hypothesis of SAR holds. High species replacement from the dispersal process is key to maintaining beta diversity. Rare species are the main contributors to species replacement, and rare species brought to habitats by the dispersal process are essential for maintaining biodiversity patterns. Coleman’s mathematical model of passive sampling is more accurate in predicting the richness of non-rare species but underestimates the abundance of rare species (especially in small habitats). Research from the perspective of speciation, dispersal, and extinction is expected to elucidate the mechanisms by which biodiversity is formed and maintained.

## Data availability statement

The raw sequence data presented in the study are deposited in the Genome Sequence Archive in National Genomics Data Center, China National Center for Bioinformation, Chinese Academy of Sciences, accession number CRA008829, that are publicly accessible at https://ngdc.cncb.ac.cn/gsa/browse/CRA008829

## Author contributions

WD, X-YY, and WX were involved in the conception and design of the study. WD and G-BY performed experimental work and collected data. WD and WX did data curation and interpretation. WD wrote the original draft of the manuscript. WX and X-YY contributed in terms of article structuring and editing. All authors contributed to the article and approved the submitted version.

## Funding

This research was funded by the Second Tibetan Plateau Scientific Expedition and Research Program (STEP), grant number 2019QZKK2002.

## Conflict of interest

The authors declare that the research was conducted in the absence of any commercial or financial relationships that could be construed as a potential conflict of interest.

## Publisher’s note

All claims expressed in this article are solely those of the authors and do not necessarily represent those of their affiliated organizations, or those of the publisher, the editors and the reviewers. Any product that may be evaluated in this article, or claim that may be made by its manufacturer, is not guaranteed or endorsed by the publisher.

## Footnotes

^1^ https://docs.qiime2.org/2019.1/
