# Supplementary material for: Testing the passive sampling hypothesis: The role of dispersal in shaping microbial species-area relationship
Source: Front Microbiol. 2023 Jan 26;14:1093695. doi: 10.3389/fmicb.2023.1093695 (PMC9909023; doi:10.3389/fmicb.2023.1093695)
Supplement: Supplementary file 2 [file Image_1.PDF]

Supplementary Material

Testing the passive sampling hypothesis: the role of dispersal on shaping microbial species-area relationship

Wei Deng, Guo-Bin Yu, Xiao-Yan Yang*, Wen Xiao*

*** Correspondence:** Xiao-Yan Yang and Wen Xiao: yangxy@eastern-himalaya.cn; xiaow@eastern-himalaya.cn

1 Supplementary Figures and Tables

1.1 Supplementary Figures

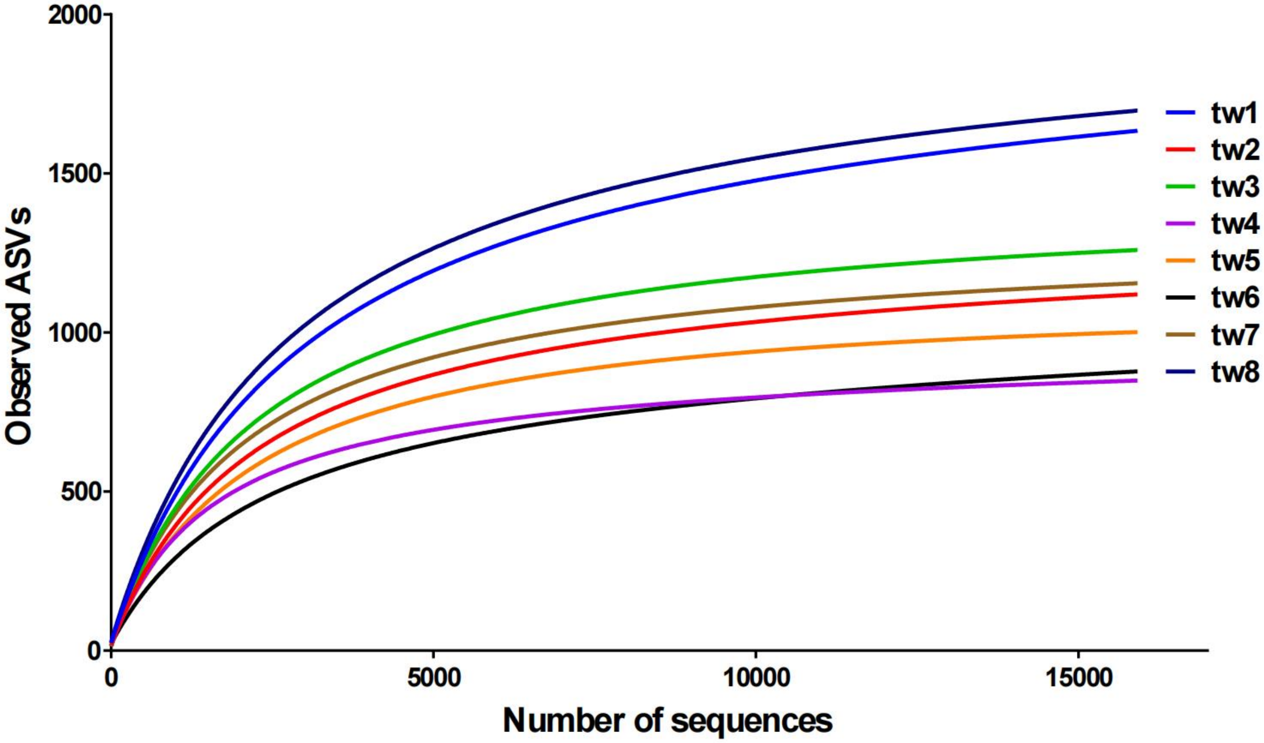

Supplementary Figure 1. Rarefaction curve of each samples. The sample ids for the filter paper are tw1 through tw8.

1.2 Supplementary Table

Supplementary Table 1 uploaded in xls format.
